# Transcriptome profiles of blastocysts originating from oocytes matured in follicular fluid from preovulatory follicles of greater or lesser maturity

**DOI:** 10.1186/s12864-025-11521-0

**Published:** 2025-04-04

**Authors:** Allyson E. Stokes, Hannah M. Clark, J. Lannett Edwards, Rebecca R. Payton, Jon E. Beever, Trevor F. Freeman, Emma A. Hessock, F. Neal Schrick, Sarah E. Moorey

**Affiliations:** Department of Animal Science, University of Tennessee Institute of Agriculture and AgResearch, 2506 River Drive, Knoxville, TN 37996 USA

**Keywords:** Blastocyst, Bovine, Cellular growth pathways, Embryo development, Follicle maturity, Metabolism, Oocyte, RNA-sequencing, Transcriptome, Wnt signaling pathway

## Abstract

**Background:**

Oocyte competence for early embryo development relies on intercellular communication between the maturing oocyte and preovulatory follicle. Preovulatory follicle maturity, as indicated by serum estradiol concentration or follicle diameter, has previously been linked to pregnancy, follicular fluid metabolites, cumulus-oocyte metabolism, and oocyte competency for embryo development. Such relationships indicate metabolic and developmental programming of the oocyte based on the preovulatory follicle’s physiological status, but downstream impacts on the molecular signature of blastocysts have not been examined. We hypothesized that supplementing maturing oocytes with follicular fluid originating from preovulatory follicles of greater or lesser maturity would impact the transcriptome of resulting blastocysts and indicate metabolic programming of the embryo that originated from the oocyte’s maturation environment. The objective was to investigate the effect of follicle maturity on the oocyte by examining the transcriptome of blastocysts originating from oocytes matured in the presence of follicular fluid from preovulatory follicles of greater or lesser maturity.

**Results:**

In vitro maturing oocytes were supplemented with follicular fluid collected from preovulatory follicles of greater or lesser maturity. Following identical embryo culture procedures, RNA-sequencing was performed on pools of 2 blastocysts (Greater, *n* = 12; Lesser, *n* = 15; all with stage code = 7 and quality code = 1). A total of 12,310 genes were identified in blastocysts after filtering to remove lowly abundant genes. There were 113 genes that differed in expression between blastocysts originating from oocytes matured in greater versus lesser maturity follicular fluid (eFDR < 0.01). Although no pathways were significantly enriched with differentially expressed genes, transcriptome profiles suggested improved Wnt/β-catenin signaling, metabolism, and protection from oxidative stress in blastocysts derived from oocytes matured in greater maturity follicular fluid, while potential unregulated cell growth presented in blastocysts resulting from the lesser follicle maturity treatment.

**Conclusions:**

Follicular fluid from preovulatory follicles of greater physiological maturity may better prepare maturing oocytes for early embryo development. Furthermore, oocytes matured in follicular fluid from preovulatory follicles of lesser maturity may attempt to overcompensate for nutrient deficit during oocyte maturation, leading to uncontrolled cellular growth and increased oxidative stress.

**Supplementary Information:**

The online version contains supplementary material available at 10.1186/s12864-025-11521-0.

## Background

Appropriate preparation of the oocyte to support early embryo development is heavily influenced by intercellular communication between the maturing oocyte and preovulatory follicle. Leading up to and during oocyte maturation, the oocyte relies on exposure to metabolites, RNA, proteins, and other substrates originating from the follicular fluid and surrounding somatic follicular cells [1–6]. Optimal oocyte competence for embryo development only occurs when the oocyte matures in vivo [7–10], and the physiological maturity of the preovulatory follicle strongly influences oocyte capacity for early embryo development [11, 12]. Initial studies investigating oocyte developmental competence utilized preovulatory follicle diameter and/or circulating estradiol (E2) concentration at the time of gonadotropin releasing hormone (GnRH) administration to induce the luteinizing hormone (LH) surge as markers of preovulatory follicle maturity. Such studies unveiled associations between follicle maturity, pregnancy, and oocyte competence for embryo development in cattle and humans alike [4, 11–18]. More recent studies in bovine have utilized RNA-sequencing and ATP analyses to reveal a distinct, positive metabolic relationship between preovulatory follicle maturity and cumulus-oocyte metabolism [19, 20]. As part of this relationship, key metabolites related to glucose and amino acid metabolism were more abundant in follicular fluid originating from preovulatory follicles of greater versus lesser maturity [3, 20].

Until recently, a causal relationship between oocyte developmental competence and the varying preovulatory follicular fluid milieu due to follicle maturity was lacking. In vitro oocyte maturation within media supplemented with follicular fluid from preovulatory follicles of greater or lesser maturity provides an intriguing technique to examine causality between preovulatory follicular fluid and oocyte metabolic competence for embryo development. Such procedures utilize pools of oocytes from similar origin; therefore, intrinsic differences in oocytes derived from follicles of greater or lesser maturity prior to oocyte maturation are removed to allow a more focused investigation of effects of an altered follicular fluid milieu on oocyte metabolism or downstream embryo development. Therefore, an in vitro study was recently performed to assess the impact of preovulatory follicle maturity on oocyte metabolism and competency for embryo development by supplementing in vitro maturing bovine oocytes with follicular fluid originating from follicles of greater or lesser physiological maturity [21]. The study revealed increased accumulation of metabolites related to glucose metabolism and other processes related to oocyte metabolic preparation in the conditioned oocyte maturation media (OMM) originally supplemented with follicular fluid from follicles of greater versus lesser maturity. These results emphasized an effect of follicle maturity on cumulus-oocyte complex (COC) metabolism and metabolic programming of the oocyte for embryo development. Blastocyst development did not differ between follicle maturity treatments, but follicle maturity was linked to differences in embryo developmental stage, as previously observed in vivo [11]. Investigation of the molecular signature of morphologically similar blastocysts produced in the study above [21] is essential to wholistically examine persisting impacts of preovulatory follicle support of the maturing oocyte. We hypothesized that supplementing follicular fluid originating from preovulatory follicles of differing physiological maturity to oocytes during in vitro maturation (IVM) would impact the transcriptome of resulting blastocysts and indicate metabolic programming of the embryo that originated from the oocyte’s maturation environment. The objective of the present study was to investigate the impact of follicle maturity on the oocyte by examining alterations in the functional genome of resulting blastocysts of similar morphology.

## Methods

### Experimental overview

**Fig. 1:**
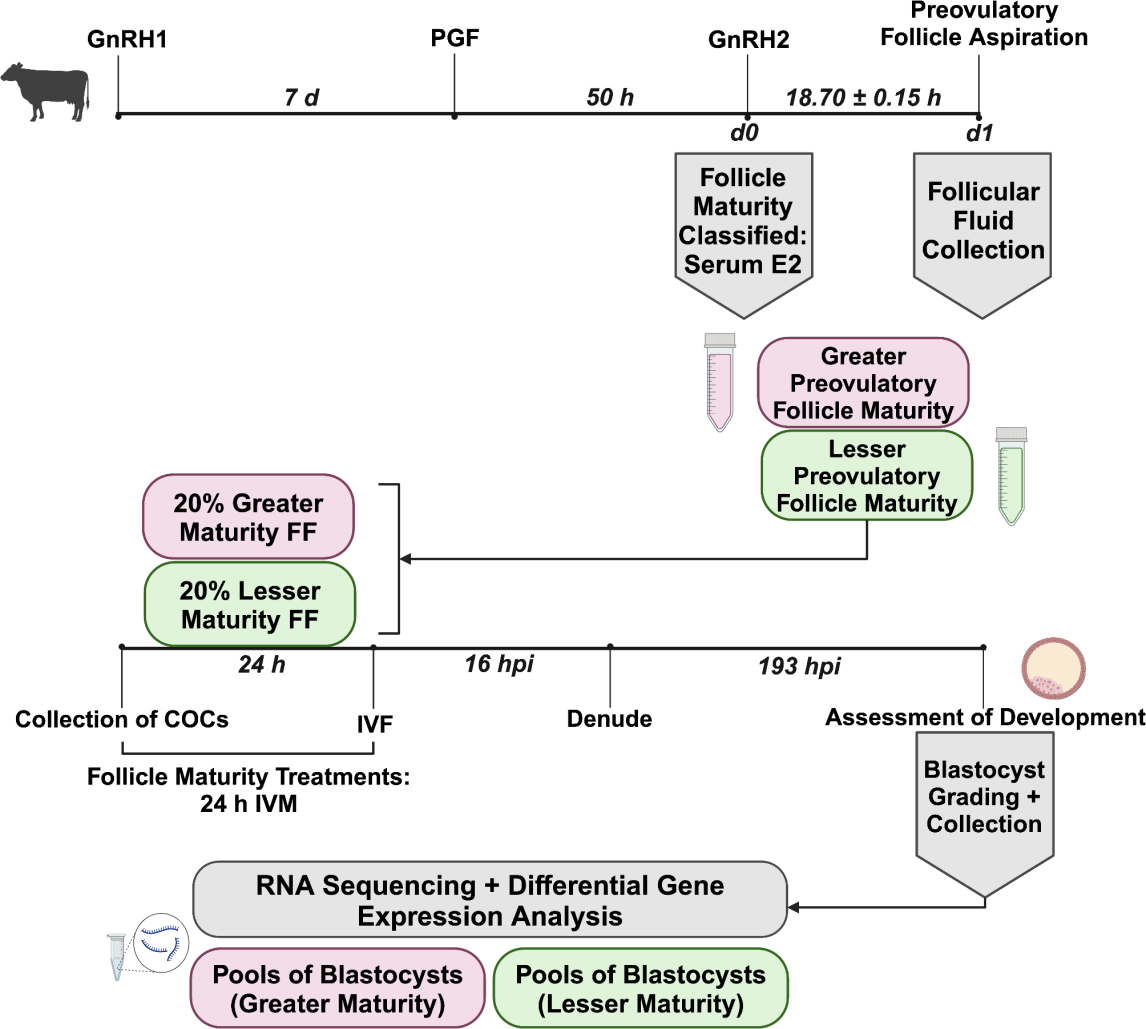
Schematic of preovulatory follicular fluid collection, follicle maturity classification, in vitro oocyte maturation, and RNA-sequencing. Preovulatory follicular fluid samples were collected 18.70 ± 0.15 h after administration of gonadotropin-releasing hormone to induce the luteinizing hormone surge (GnRH2). Serum estradiol (E2) concentration at GnRH2 was used to classify follicles as greater or lesser physiological maturity. Immature bovine cumulus-oocyte complexes (COCs) were randomly assigned to either greater or lesser follicle maturity treatments. Following 24 h oocyte maturation (24 h IVM), in vitro fertilization (IVF) was performed. At 193 h post IVF (hpi), blastocysts were staged, graded, and collected. Aqueous phases of 2 blastocysts from the same replicate and treatment were pooled during RNA extraction. RNA-sequencing libraries were prepared and sequenced. Differences in gene expression were compared between blastocysts originating from oocytes of the greater and lesser follicle maturity treatments. GnRH1, gonadotropin-releasing hormone first administration; PGF, prostaglandin F2α

Follicular fluid samples were collected from bovine preovulatory follicles approximately 18 hours after GnRH was administered to induce the LH surge [20]. Samples were then categorized into greater and lesser maturity classifications based on serum E2 levels at GnRH administration. Immature oocytes were matured in vitro for 24 hours in OMM supplemented with 20% follicular fluid of greater or lesser follicle maturity, and COCs of each treatment underwent identical procedures for fertilization, denudement, and embryo culture for 8 days [21]. Blastocysts were collected after completion of staging and grading on day 8. Total RNA was extracted from 33 pools of 2 expanding or expanded, grade 1 blastocysts, and 3’ mRNA libraries were prepared and sequenced. The transcriptome of greater versus lesser maturity blastocysts was then compared using edgeR and DESeq2 (Fig. 1).

### Synchronization of a preovulatory follicle and follicular fluid collection

All procedures involving animals utilized animals owned by the University of Tennessee AgResearch and were approved by the University of Tennessee Institutional Animal Care and Use Committee. Animals were not euthanized or sacrificed as a part of this experiment and returned to normal daily activities after the completion of this project. Preovulatory follicles were synchronized in postpartum, lactating Angus cows (*n* = 136) using a presynchronization followed by a 7-Day CO-Synch protocol described by Read et al. [3] (Fig. 1). Gonadotropin-releasing hormone (GnRH; 100 µg Cystorelin, Boehriner Ingelheim, Ingelheim am Rhein, Germany) was administered to induce the preovulatory LH surge approximately 50 h after prostaglandin F2 alpha (PGF; 25 mg Lutalyse^®^ HighCon, Zoetis Animal Health) administration. Transvaginal aspiration to collect preovulatory follicular fluid samples (*n* = 103) was performed 18.70 ± 0.15 h after administration of GnRH. This timepoint was chosen due to previous research which showed negative impacts on oocyte maturation and blastocyst development after in vitro maturation supplementation of follicular fluid collected 0 h after GnRH administration compared to 20 h after [22]. The follicular fluid was transferred into a 1.7 mL tube (VWR, Radnor, PA, USA) and centrifuged at 500 x g at 4° C for 5 min to pellet any cellular debris. Follicular fluid samples were snap frozen and stored at -80° C.

To ensure preovulatory follicular fluid samples were collected from cows relying on exogenous GnRH administration to induce a preovulatory gonadotropin surge, Estrotect™ patches were utilized for identification and removal of animals that displayed estrus at any timepoint during synchronization. Patches were scored on a scale of 0 to 4 to indicate a missing patch (patch = 0), < 25% rubbed off (patch = 1; no estrus), 25–50% rubbed off (patch = 2; no estrus), 50–75% rubbed off (patch = 3; estrus), and > 75% rubbed off (patch = 4, estrus).

### Preovulatory follicle maturity classification and in vitro embryo production

Preovulatory follicular fluid treatments were those utilized in Clark et al. 2024 [21]. Briefly, serum E2 concentrations at GnRH2 were used to classify follicular fluid samples into upper, intermediate, and lower thirds (Fig. 1). Follicle dynamics and circulating hormone profiles were assessed, and only samples corresponding to a growing, estrogen active follicle prior to GnRH2 were utilized. Any follicular fluid containing blood, contaminants, samples with < 100 µL in volume, or samples with estradiol: progesterone (E2:P4) ratio > 3.12 (*n* = 43) were removed [23–25]. The remaining samples were classified accordingly with the upper third (serum E2: 9.5–15.4 pg/mL) becoming the greater preovulatory follicle maturity treatment (*n* = 17) and the lower third (serum E2: 1.9–4.9 pg/mL) becoming the lesser preovulatory follicle maturity treatment (*n* = 12, Fig. 1). Follicular fluid samples from each follicle maturity category were pooled to form a homogenous aliquot for single thaw use during each replicate of in vitro oocyte maturation.

Ovaries utilized in this study were sourced from a local abattoir who processes predominantly Angus, Holstein, Hereford, Simmental, and Charolais cattle. In vitro production of embryos was performed in 11 replicates as part of a larger study [21] using procedures described in Edwards et al. [26] and Rispoli et al. [27]. For each replicate, COCs were collected from the ovary using a scalpel to create a crosshatch pattern across antral follicles (~ 2–8 mm). Recovered COCs with dark, evenly granulated ooplasm and at least 3 layers of cumulus cells were randomly grouped in consistent numbers of approximately 30 COCs and assigned to prepared oocyte maturation plates (4-well culture dish). Each well contained 500 µL Medium-199 without phenol red (OMM) that was supplemented with Earle’s salts, 50 µg/mL gentamicin, 5.0 µg/mL follicle stimulating hormone (FSH), 0.3 µg/mL LH, 0.2mM sodium pyruvate, 2mM l-glutamine, and either 20% follicular fluid from preovulatory follicles of greater follicle maturity or 20% follicular fluid from preovulatory follicles of lesser follicle maturity. Per each replicate, one plate with three wells of COCs per follicular fluid treatment were then placed in a 38.5° C, 5.5% CO_2_, 21% O_2_ environment to mature for 24 h.

After 24 h of IVM, COCs from 1 well were collected for metabolic analyses outside the scope of the current study and COCs from the remaining 2 wells underwent in vitro fertilization (IVF) with Percoll-prepared frozen-thawed semen of the same bull (500,000 motile sperm/mL). Approximately 16 h post-IVF (hpi), putative zygotes (PZ) were denuded of cumulus cells and spermatozoa by vortexing for 4.5 min in stock solution containing HEPES-TALP and 1 mg/mL hyaluronidase. Putative zygotes were then cultured in 500 µL of potassium supplemented simplex optimized medium (KSOM) at 38° C, 5.5% CO_2_, 7.0% O_2_.

Blastocyst development rate was assessed approximately 193 hpi (Day 8), and stage and quality scores were assigned to resulting embryos according to the International Embryo Technology Society (IETS) guidelines [27–29]. Day 8 embryos were categorized as either compact morula (Stage Code 4), early blastocyst (Stage Code 5), normal blastocyst (Stage Code 6), expanding or expanded blastocyst (Stage Code 7), and hatching or hatched blastocyst (Stage Code 8). Quality codes for the embryos were also assigned based on the IETS Manual guidelines of excellent or good (Code 1), fair (Code 2), poor (Code 3), and dead or degenerating (Code 4).

### Sample collection, RNA extraction, library preparation, and RNA sequencing

Following the completion of development assessments on Day 8, all individual blastocysts from the first 4 of the 11 total replicates were placed into 0.2 mL RNase free collection tubes with 2 µL of HEPES-TALP and 75 µL of TRIzol^®^ reagent, snap frozen in liquid nitrogen, and stored at -80° C until RNA extraction. The blastocysts from the remaining 7 replicates were collected and stored for use in a future study outside the scope of this manuscript. There was advanced embryo stage in embryos produced from oocytes matured in the presence of follicular fluid from follicles of lesser versus greater maturity when considering all embryos produced in Clark et al. [21]. The current study, however, utilized only blastocysts of similar morphology to allow a more focused and controlled investigation of the differences in the blastocyst transcriptome due to follicle maturity treatment during oocyte maturation.

Therefore, total RNA was isolated from 33 pools of 2 blastocysts with stage score 7 and quality score 1 (IETS) [greater follicle maturity (*n* = 16), lesser follicle maturity (*n* = 17)] using an optimized TRIzol^®^ protocol [30]. Single blastocyst samples were pooled during RNA extraction by combining aqueous phases from 2 samples of the same treatment and replicate before RNA precipitation. TapeStation 4200 (Agilent Technologies) was used to evaluate RNA concentration and quality. Approximately 250 pg total RNA was used as input for reverse transcription, and cDNA amplification was performed using the SMART-Seq v4 3’ DE Kit following the manufacturer’s protocol (Takara Bio) with volumes adjusted to complete procedures using half reactions. Resulting cDNA was then assessed using TapeStation 4200 (Agilent Technologies), and approximately 500 pg cDNA was utilized for library preparation using the Illumina Nextera XT DNA Sample Preparation Kit and following the manufacturer’s protocol (Illumina). Libraries were pooled in equal concentration and sequenced on an Illumina NovaSeq 6000 using an S4 200-cycle flow cell to generate 1 × 150 (forward, R1) and 1 × 28 (reverse, R2) paired-end reads at a sequencing depth of approximately 30 million reads per sample (Additional file 1).

### RNA sequencing data processing

Only forward (R1) reads were used as input for data processing since reverse (R2) reads from the SMART-Seq v4 3’ DE Kit are not informative beyond indexing. Quality of raw reads was assessed using FastQC [31] with subsequent read trimming and filtering using fastp [32]. Trimmed reads were then reassessed using FastQC and mapped to the bovine genome (ARS-UCD2.0) using STAR [33]. Alignments were sorted and indexed with SAMtools [34], and alignment quality control was performed with QualiMap [35]. The featureCounts tool from SubRead [36] was then used to count reads.

### RNA sequencing statistical analyses

All statistical analyses were conducted using RStudio. Of the original 33 blastocyst RNA pools, 6 pools were not included in our statistical analyses due to insufficient read depth following sequencing (2 greater maturity pools from replicates 1 and 4; 1 lesser maturity pool from replicates 1 and 4). Counts per million (CPM) were calculated and transcripts were filtered to retain only those with at least 1 CPM in 12 blastocyst pools. MetaboAnalyst 5.0 was utilized to conduct orthogonal partial least squares (OPLS) analysis with the resulting filtered list of transcripts. Bioconductor packages “edgeR” [37] and “DESeq2” [38] were used for statistical analyses between the greater follicle maturity (*n* = 12) and lesser follicle maturity (*n* = 15) blastocyst pools, with models designed to account for both follicle maturity and replicate. Empirical false discovery rate (eFDR) was then calculated using 10,000 permutations of sample reshuffling outlined elsewhere [39–41]. Genes were considered differentially expressed between follicle maturity treatments if eFDR was ≤ 0.01 in both “edgeR” and “DESeq2” analyses [19, 42–44]. The resulting differentially expressed gene list was uploaded into the Database for Annotation, Visualization, and Integrated Discovery (DAVID) program to assess enrichment of KEGG pathways, gene ontology (GO) biological processes, GO cellular components, or GO molecular functions [45]. Significance was determined if FDR < 0.05.

## Results

### Overview of RNA sequencing data

**Fig. 2:**
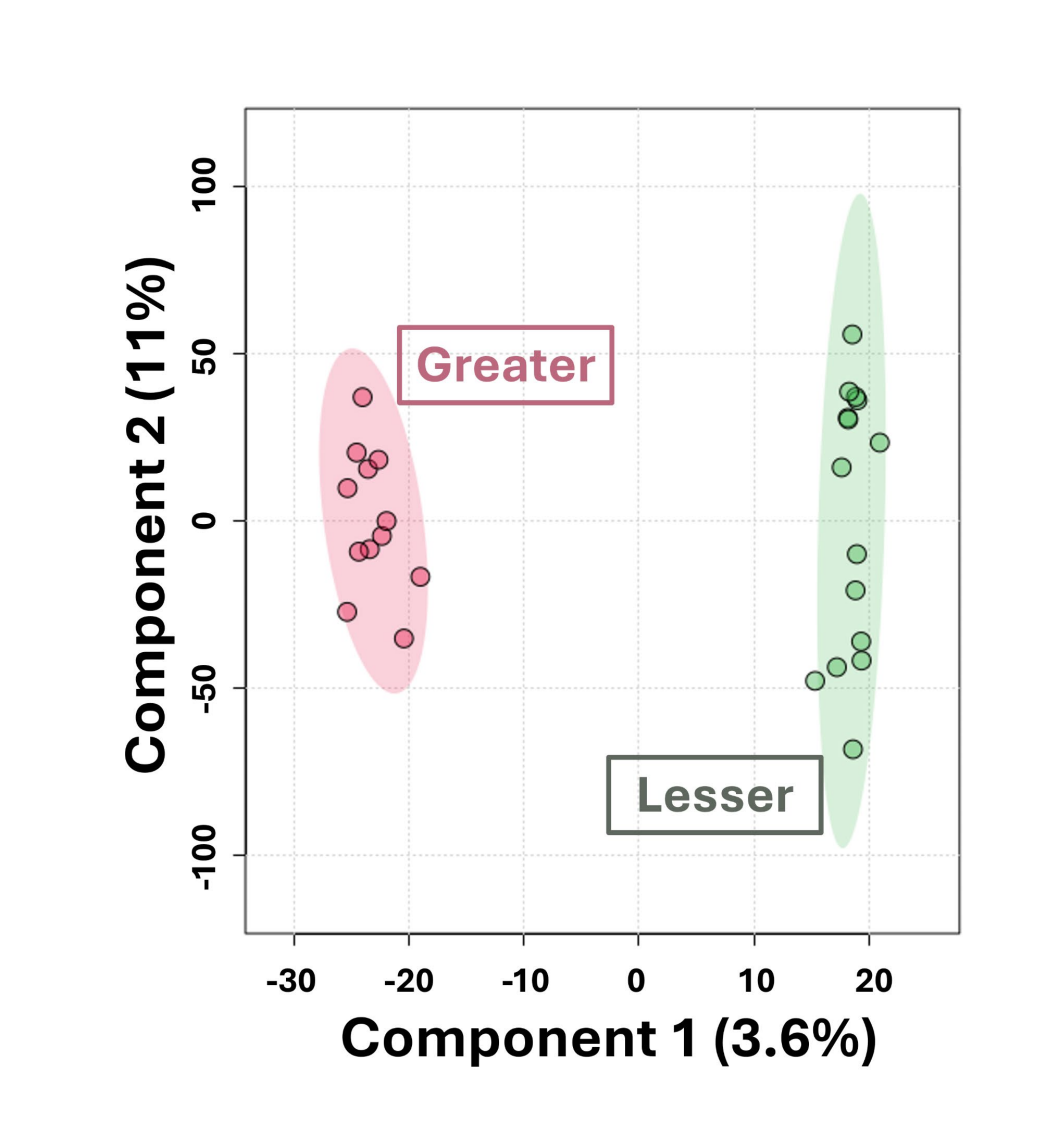
Orthogonal partial least squares analysis of transcriptome data from greater and lesser maturity blastocysts

Transcriptome data was generated from in vitro produced bovine blastocysts resulting from oocytes supplemented with follicular fluid from preovulatory follicles of greater (*n* = 12) or lesser (*n* = 15) maturity during IVM. An average of 29.5 million reads per sample was produced from sequencing, with an average of 28.5 million mapped to the bovine genome (96.4%, Additional file 1). A total of 12,310 genes were used for analyses of differential gene expression following filtering of lowly expressed genes. The OPLS analysis clustered samples based on preovulatory follicle maturity treatment, but only 3.6% of the variation among samples was explained by follicle maturity (Fig. 2).

### Differentially expressed genes between greater and lesser follicle maturity blastocysts

One hundred thirteen genes differed in expression between quality score 1, expanding or expanded blastocysts produced from oocytes exposed to follicular fluid from preovulatory follicles of greater versus lesser maturity during IVM (Additional file 2). Thirty-seven of the follicle maturity related genes were more abundant in blastocysts of the greater follicle maturity treatment while 76 were more abundant in blastocysts resulting from oocytes supplemented with follicular fluid of lesser physiological maturity. No pathways or gene ontologies were significantly enriched with differentially expressed genes.

**Fig. 3:**
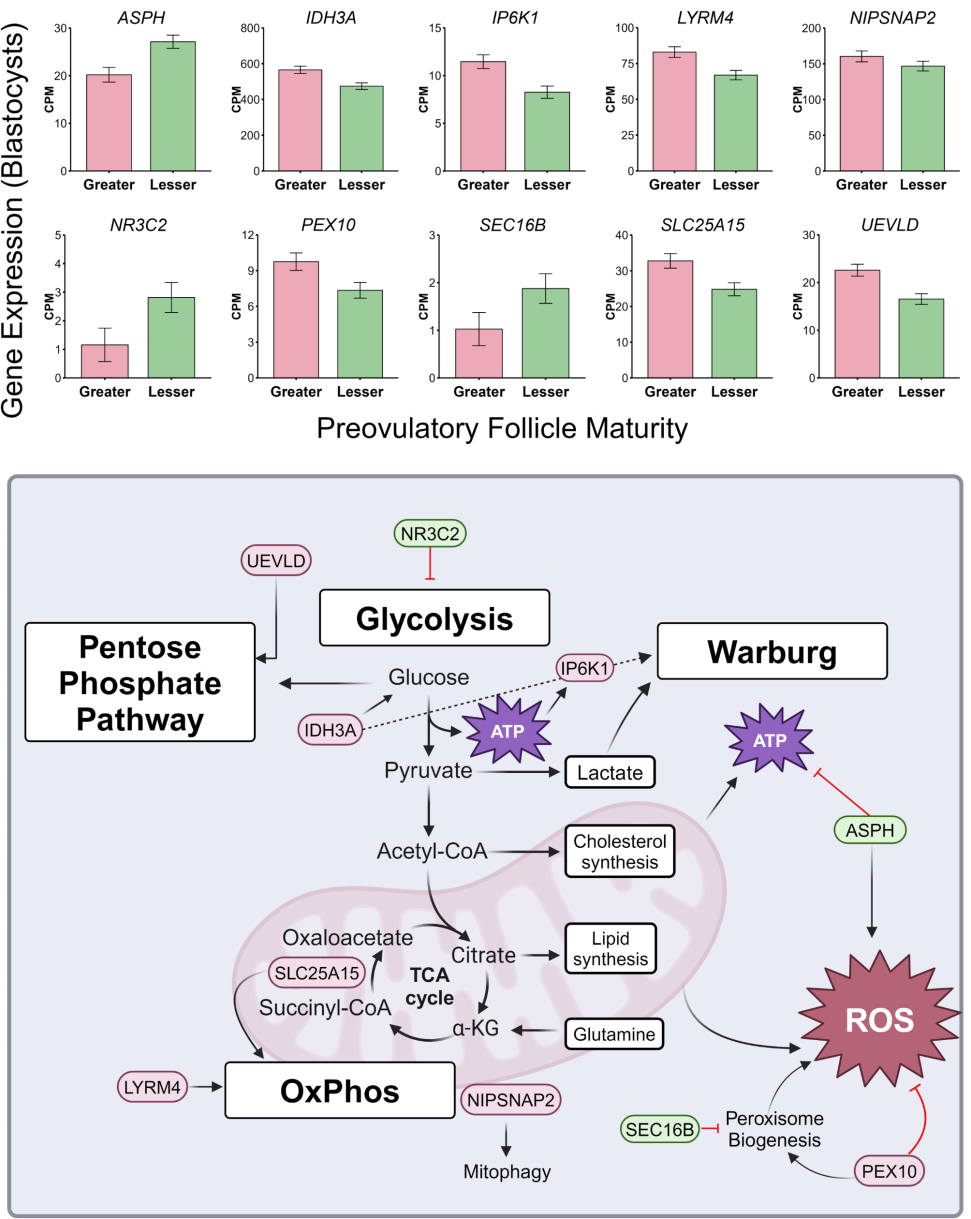
Genes involved in glucose metabolism, mitochondrial function, and oxidative stress that differed in expression between blastocysts resulting from oocytes supplemented with follicular fluid of preovulatory follicles of greater or lesser maturity during in vitro oocyte maturation. Bar graphs display counts per million (CPM) of each gene in blastocysts from greater and lesser follicle maturity treatments. Schematic displays gene association with or action on various metabolic processes (created using Biorender.com)

A total of 10 genes (9%) that have noted involvement within metabolic processes (i.e. glycolysis), mitochondrial function, and oxidative stress were differentially abundant between follicle maturity treatments (Fig. 3). Genes indicative of active glycolysis (*IDH3A*,* IP6K1*), glucose bypass to the pentose phosphate pathway (*UEVLD*), mitochondrial function (*LYRM4*,* NIPSNAP2*,* SLC25A15*), and peroxisome biogenesis or mitigation of oxidative stress (*PEX10*) had greater mRNA abundance in blastocysts originating from the greater follicle maturity treatment. Alternatively, genes known to be associated with elevated reactive oxygen species (ROS) and decreased mitochondrial activity for ATP production (*ASPH*), inhibit glycolysis (*NR3C2*), or reduce bioavailability of peroxisomes (*SEC16B*) had greater mRNA abundance in blastocysts produced from oocytes matured in the presence of follicular fluid from preovulatory follicles of lesser maturity.

**Fig. 4:**
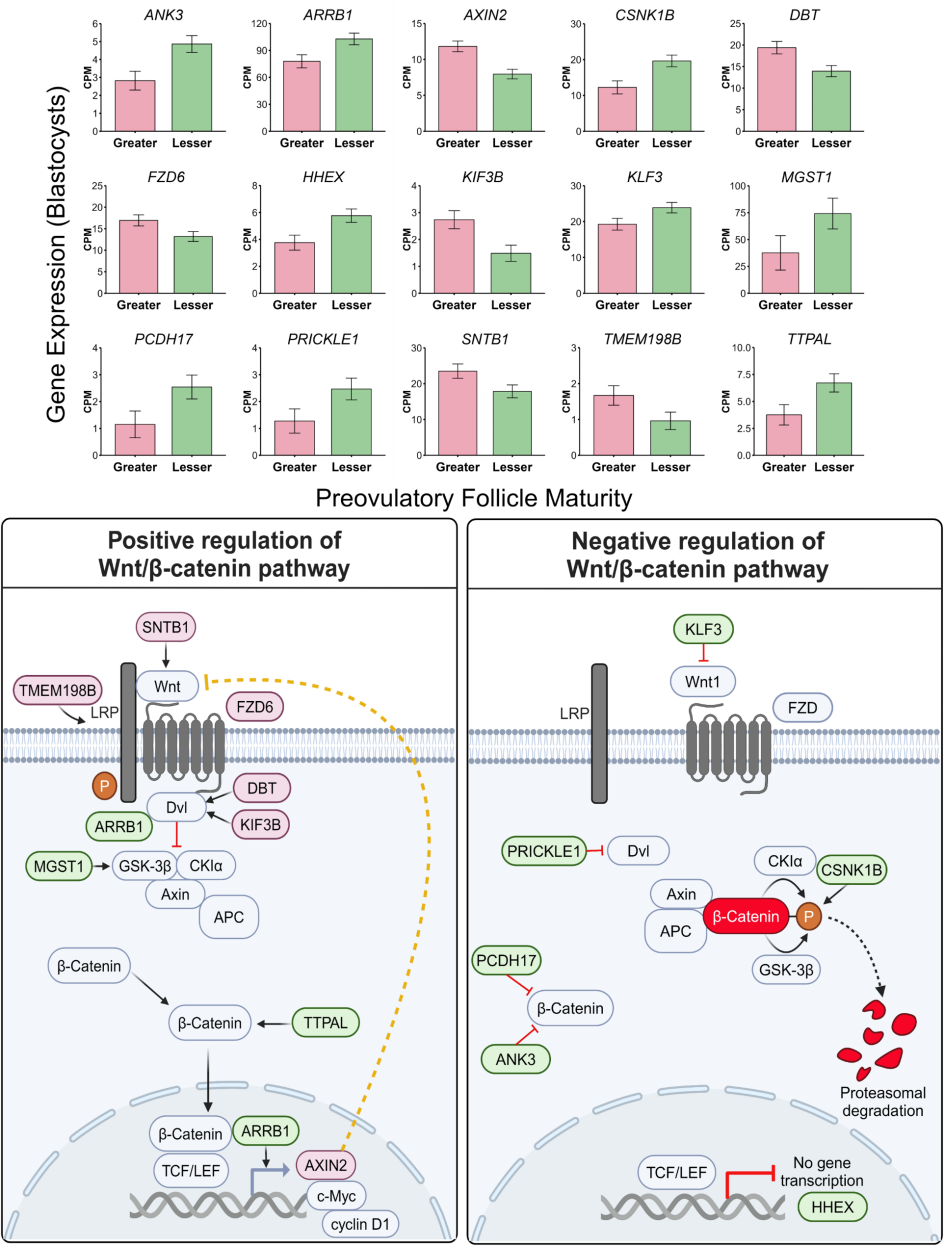
Genes involved in positive or negative regulation of the Wnt/β-catenin signaling pathway that differed in expression between blastocysts resulting from oocytes supplemented with follicular fluid of preovulatory follicles of greater or lesser maturity during in vitro oocyte maturation. Bar graphs display counts per million (CPM) of each gene in blastocysts from greater and lesser follicle maturity treatments. Schematic displays gene association with or action on various steps in the Wnt/β-catenin signaling pathway (created using Biorender.com)

Fifteen (13%) of the differentially expressed genes between blastocysts from oocytes in the greater versus lesser follicle maturity treatment were involved in the Wnt/β-catenin signaling pathway. All Wnt/β-catenin related genes with greater expression in greater follicle maturity blastocysts had a positive role in stimulating the pathway (*DBT*,* FZD6*,* KIF3B*,* SNTB1*,* TMEM198B)* or were a target gene whose transcription is stimulated by nuclear β-catenin signaling (*AXIN2*; Fig. 4*).* Alternatively, while *ARRB1*,* MGST1*, and *TTPAL* which positively regulate the Wnt/β-catenin signaling pathway were more abundant in blastocysts from the lesser follicle maturity treatment, all other genes with greater abundance in blastocysts resulting from oocytes matured in vitro in the presence of lesser maturity follicular fluid hold functions of negative regulation on the Wnt/β-catenin pathway (*ANK3*,* CSNK1B*,* HHEX*,* KLF3*,* PCDH17*,* PRICKLE1*; Fig. 4).

**Fig. 5:**
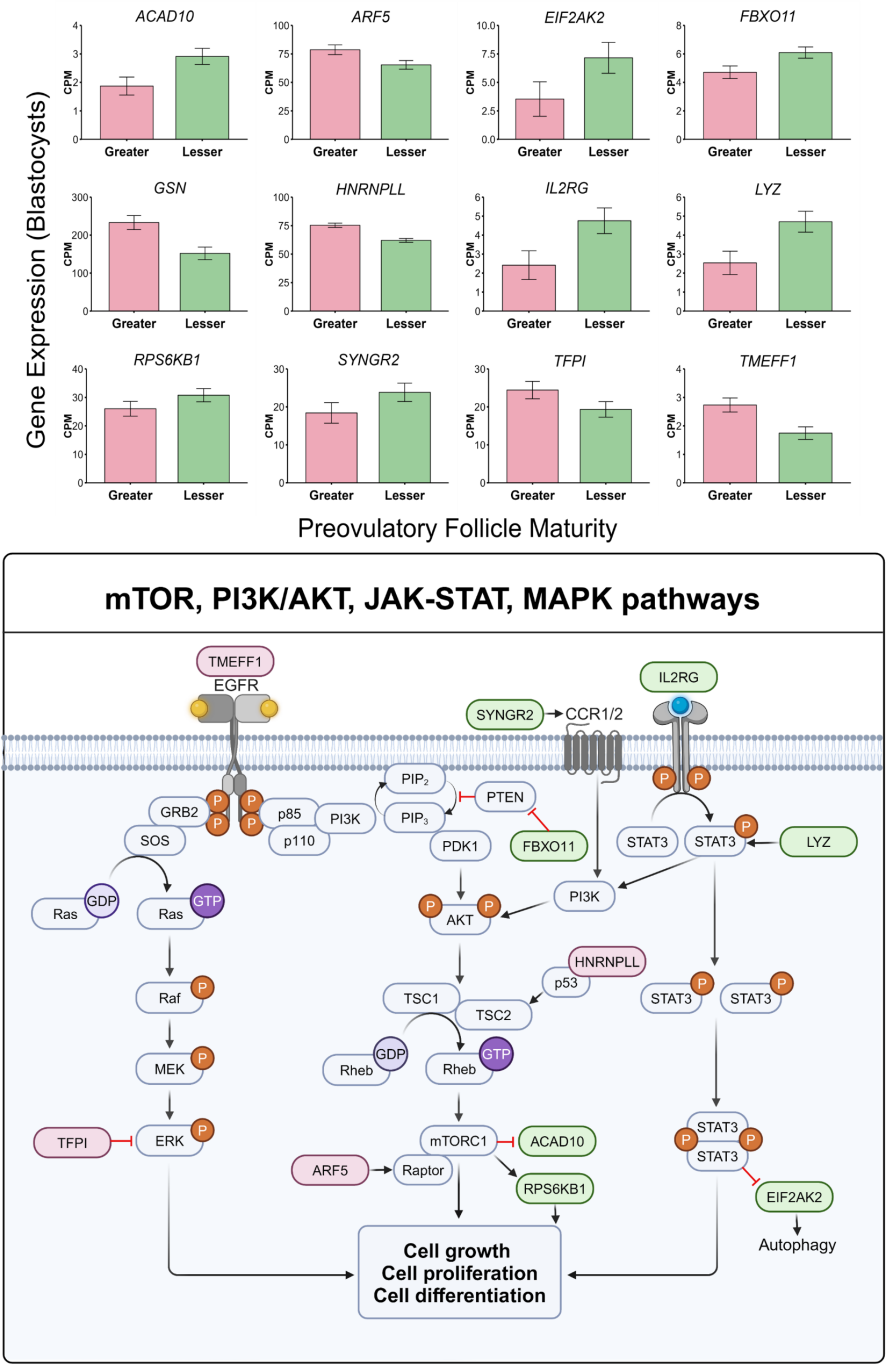
Genes involved in cell growth and division related pathways of mTOR, PI3K/AKT, MAPK, and JAK-STAT that differed in abundance between blastocysts resulting from oocytes supplemented with follicular fluid of preovulatory follicles of greater or lesser maturity during in vitro oocyte maturation. Bar graphs display counts per million (CPM) of each gene in blastocysts from greater and lesser follicle maturity treatments. Schematic displays gene association with or action on various steps in the cell growth signaling pathways (created using Biorender.com)

Finally, 12 (11%) of the differentially expressed genes between blastocysts produced from oocytes supplemented with follicular fluid of preovulatory follicles of greater of lesser physiological maturity were associated with cellular growth and division related pathways including mammalian target of rapamycin (mTOR), phosphoinositide 3-kinase/v-AKT murine thymoma viral oncogene homolog 1 (PI3K/AKT), mitogen-activated protein kinase (MAPK), and Janus-activated kinase/signal transducer and activator of transcription (JAK/STAT; Fig. 5). Five of such genes had greater expression in blastocysts of the greater follicle maturity treatment (*ARF5*,* GSN*,* HNRNPLL*,* TFPI*,* TMEFF1*), while 7 had greater expression in blastocysts derived from oocytes exposed to preovulatory follicular fluid from the lesser follicle maturity treatment (*ACAD10*,* EIF2AK2*,* FBX011*,* IL2RG*,* LYZ*,* RPS6KB1*,* SYNGR2*).

## Discussion

Results demonstrate an effect of preovulatory follicle support of the maturing oocyte that persists throughout early embryo development to impact the molecular signature of blastocysts with similar morphology. Transcriptome profiles of blastocysts originating from oocytes supplemented with follicular fluid from greater maturity follicles during in vitro maturation indicated potentially improved metabolism and cell signaling through the Wnt/β-catenin pathway. Alternatively, results suggest unregulated cell division, similar to that in cancer cells, and potential oxidative stress in blastocysts originating from oocytes supplemented with lesser follicle maturity follicular fluid. Blastocysts in the current study were generated as part of a separate study to determine the impact of preovulatory follicle maturity on oocyte developmental and metabolic competence for embryo development [21]. Although there was no difference in blastocyst development between oocytes matured in the presence of follicular fluid from greater (26.2%) or lesser (24.8%) maturity preovulatory follicles (*p* = 0.28) [21], results from both Clark [21] and an in vivo study assessing the relationship between preovulatory follicle maturity and embryo quality [11] demonstrated a more moderate rate of embryonic development in oocytes matured in the follicular fluid of preovulatory follicles of greater versus lesser physiological maturity. To remove confounding impacts of embryo stage and quality on the blastocyst transcriptome, the current study utilized only blastocysts of stage score 7 and quality score 1. Results within, therefore, examine morphologically similar embryos and highlight small differences in the transcriptome due to oocyte maturation environment that may impart biological relevance for future conceptus development and pregnancy success. Taken together with profound differences in follicular fluid metabolite profile based on preovulatory follicle maturity [3, 20], the RNA-sequencing results of the present study lead us to hypothesize that the follicular fluid environment of preovulatory follicles of greater maturity provides a metabolically ideal environment for the maturing oocyte that programs the oocyte for energy efficient metabolism and cellular divisions during early embryo development.

Profound differences in composition were observed in preovulatory follicular fluid of greater or lesser maturity [3, 20], and in the follicular fluid supplemented media in which oocytes were matured in to generate blastocysts utilized in the current study [21]. Although we did not measure if metabolite transfer from the cumulus cells to the oocyte was increased due to differing metabolite abundance between treatments, it has been documented that low amino acid composition in media compromises the transfer of reagents from the external environment through the cumulus cells and into the oocyte [46]. Metabolomics prior to onset of oocyte maturation showed an increase in abundance of 40 metabolites in the greater versus lesser follicle maturity supplemented media with pathway analysis revealing enrichment of “Arginine biosynthesis” and “Purine metabolism” pathways with such metabolites [21]. One could speculate that the higher abundance of numerous metabolites, particularly amino acids, from follicular fluid of greater follicle maturity, and the resulting media supplemented with greater maturity follicular fluid, increased transfer between cumulus cells and maturing oocytes. No previous research has documented that additional metabolite bioavailability in the maturing oocyte leads to storage for embryo development; however certain protein complexes can be stored and are important for epigenetic reprogramming in the embryo [47]. Metabolites such as methionine, which was more abundant in greater maturity follicular fluid [3] and had a positive change in abundance in media after oocyte maturation in greater follicle maturity follicular fluid [21], may also set the stage for epigenetic modulations of the oocyte that impact embryo gene expression leading to differential profiles observed in the current study.

Considerable differences in metabolism due to follicle maturity treatment were observed in the maturing oocytes that became blastocysts utilized in the current study [21]. Transcriptome profiles of these resulting blastocysts suggest that metabolic programming of the oocyte persisted to the early embryo as metabolic gene expression suggested increased capacity for glucose uptake, glycolysis, and mitochondrial function in blastocysts of the greater follicle maturity treatment (Fig. 3). Abundance of isocitrate dehydrogenase 3-alpha (IDH3A), which was greater expressed in the blastocysts resulting from oocytes matured in the presence of preovulatory follicular fluid from follicles of greater maturity, has previously been correlated with glucose uptake [48]. Considering this, it is interesting that conditioned embryo culture media from embryos resultant of oocytes exposed to greater maturity follicular fluid contained more glucose phosphate than the lesser follicle maturity treatment [21]. This said, it is difficult to directly connect the metabolite usage or production in conditioned embryo culture media to the results of the current study, as all developing embryos in each well of media contributed to its metabolite milieu. One possibility for increased glucose phosphate in the conditioned embryo development media of the greater maturity treatment is that blastocysts of advanced stage from the lesser maturity treatment required additional glucose to fuel their hastened cell division and greater overall number of cells.

Also related to metabolism, the enzyme inositol hexakisphosphate kinase 1 (IP6K1), which was also greater expressed in the greater follicle maturity blastocysts, functions in the conversion of inositol hexakisphosphate to generate downstream factors important for cellular energy, glycolysis, and mitochondrial function [49]. As a measure of glycolytic activity, lactate is one of the primary products of glycolysis, and the enzyme lactate dehydrogenase A (LDHA) helps promote the conversion of pyruvate to lactate at the end of the glycolytic pathway [50]. A gene encoding an enzyme similar to lactate dehydrogenases, UEV and lactate/malate dehydrogenase domains (*UEVLD*), which may promote oxidoreductase activity to control the bypass of glycolysis for the pentose phosphate pathway [51], had higher mRNA abundance in blastocysts from the greater follicle maturity treatment. Alternatively, overexpression of nuclear receptor subfamily 3 group C member 2 (NR3C2), which had greater expression in blastocysts resulting from oocytes matured in follicular fluid of preovulatory follicles of lesser maturity, has been implicated with inhibition of glucose metabolism by decreasing overall expression of hexokinase 2 (HK2) and LDHA [52].

In addition to glycolysis, multiple differentially expressed genes involved in various mitochondrial mechanisms were noted in the current study, including *LYRM4*, *NIPSNAP2*, *PEX10*, and *SLC25A15*, all of which were higher in abundance in blastocysts resulting from oocytes matured within follicular fluid from preovulatory follicles of greater maturity (Fig. 3). Proteins encoded by both *LYRM4* and *NIPSNAP2* play critical roles in mitochondrial function with that of *LYRM4* supporting downstream oxidative phosphorylation components [53] and *NIPSNAP2* signaling autophagy receptors to translocate to the outer mitochondrial membrane for regulated mitophagy [54]. Additionally, members of the mitochondrial carrier family of proteins (solute carrier family 25; SLC25) are involved in several metabolic processes, including the citric acid cycle and oxidative phosphorylation [55]. Member 15 of the SLC25 family (SLC25A15), which had greater expression in blastocysts originating from oocytes from the greater follicle maturity treatment, has been established as a mitochondrial ornithine carrier [56], which could indicate increased transport of essential substrates for developing blastocysts resulting from oocytes metabolically supported by follicular fluid of preovulatory follicles of greater maturity during oocyte maturation.

Energy production inevitably leads to production of ROS. Physiologically, ROS can promote cellular proliferation and survival while also inducing apoptosis and DNA damage at increased concentrations [57, 58]. Increased production of ROS without appropriate defense mechanisms during embryo development can also negatively impact mitochondrial function [59]. Overexpression of aspartate β-hydroxylase (ASPH), whose mRNA was more abundant in blastocysts from the lesser follicle maturity treatment, has been linked to both enhanced intracellular ROS as well as decreased mitochondrial function and subsequent lower ATP [60]. Towards protection from excessive ROS, peroxisomal biogenesis factor 10 (PEX10) is a critical component involved in ROS detoxification through peroxisome maintenance. This protein may be exerting a protective factor for developing blastocysts resultant of oocytes exposed to follicular fluid from physiologically mature follicles since its gene’s expression was higher in blastocysts of the greater follicle maturity treatment. Alternatively, peroxisome functionality may be reduced in blastocysts from the lesser follicle maturity treatment, as indicated by higher abundance of *SEC16B*, since this gene’s overexpression led to a loss of peroxisomes in previous studies [61, 62]. Increased expression of genes related to cellular proliferation, unregulated cellular divisions, and increased abundance of *GSTA1*, a gene encoding a mitochondrial enzyme induced by increased stress conditions, force one to consider that increased cellular proliferation and growth rates could possibly be contributing to increased oxidative stress in the lesser follicle maturity blastocysts.

One limitation of the current study is that embryos were not transferred to assess developmental competency for pregnancy. As such, no conclusions about embryo competency to establish pregnancy can be made from the transcriptome results reported here. The metabolic implications described above combined with compelling transcriptome profiles related to Wnt signaling in the greater follicle maturity treatment, however, suggest greater embryo competence when oocytes are supplemented with follicular fluid from preovulatory follicles of greater physiological maturity. During embryo development, Wnt signaling pathways regulate cell growth processes [63–65]. One notable finding of the current study was that mRNA levels of genes encoding proteins essential for the Wnt/β-catenin signaling pathway suggested a more functional pathway with potential for greater intracellular β-catenin bioavailability in blastocysts of the greater follicle maturity treatment (Fig. 4). Greater expression of genes for both the G-protein coupled transmembrane frizzled receptor (*FZD6*) and associated transmembrane protein 198B (*TMEM198B*) suggest a more functional receptor complex to initiate the Wnt signaling pathway in blastocysts resulting from oocytes exposed to follicular fluid of preovulatory follicles with greater maturity. Additionally, greater abundance of the genes *DBT*, *KIF3B*, and *SNTB1*, whose proteins promote functionality of disheveled (Dvl) and its inhibition of the β-catenin destruction complex [66], implies improved Wnt signaling cascades and elevated intracellular β-catenin in blastocysts originating from oocytes in the greater maturity treatment. Indeed, Wang et al. [67] observed that overexpression of KIF3B led to increased availability of β-catenin and increased expression of target genes (i.e. cyclin D1, c-Myc), while silencing of KIF3B downregulated β-catenin. Further, cellular growth via Wnt/β-catenin was suppressed [68], and Wnt1 levels as well as c-Myc and cyclin D1 transcription were downregulated when SNTB1 was silenced [69].

Furthermore, *CSNK1B*, *PRICKLE1*, *ANK3*, and *PCDH17*, all of which encode proteins that reduce bioavailability of β-catenin, had higher abundance in blastocysts from the lesser follicle maturity treatment (Fig. 4). The protein encoded by *CSNK1B* is an isoform of the destruction complex component, casein kinase that has been demonstrated to promote degradation of β-catenin and prevent downstream transcription [70]. Also related to potentially reduced β-catenin availability in blastocysts of the lesser follicle maturity treatment, ANK3 functionality has been connected to reduced β-catenin availability in proliferating cells [71], and the protein encoded by *PRICKLE1* promotes Dvl degradation [72], thereby removing its negative regulatory functions on the β-catenin destruction complex [65]. Protocadherins are critical regulatory components for cellular processes such as adhesion and signaling pathways [73]. However, some protocadherins encode tumor suppressor genes (TSG), including *PCDH17* [74, 75] which has previously been shown to act as an inhibitor of Wnt/β-catenin signaling by reducing β-catenin and downstream mRNA expression [76].

In addition to genes directly related to reduced β-catenin availability, lower abundance of *KLF3* in blastocysts of the greater follicle maturity treatment suggests increased activation of Wnt signaling. Shen et al. [77] conducted a study demonstrating that depletion of KLF3 protein may lead to increased activation of Wnt/β-catenin signaling through upregulation of the Wnt1 ligand. It is essential to note that 4 genes that were greater expressed in the lesser follicle maturity treatment have positive forms of regulation on Wnt/β-catenin (Fig. 4). Furthermore, transcriptome data in the current manuscript does not directly measure protein abundance. This said, patterns of abundance observed in the majority of Wnt regulatory genes in blastocysts of the greater or lesser follicle maturity treatment, and higher abundance of *AXIN2* in the greater follicle maturity treatment, which is a β-catenin target gene that is upregulated by functional Wnt/β-catenin signaling [78], provide a compelling case that blastocysts from the greater follicle maturity treatment may exhibit higher bioavailability of β-catenin and more functional Wnt signaling than their lesser follicle maturity counterparts.

Aside from Wnt/β-catenin signaling, the functional mechanisms of mTOR, PI3K/AKT, MAPK, and JAK/ STAT signaling pathways are equally critical for cell growth and survival [79, 80]. Evidence likewise indicates that crosstalk between these various pathways is beneficial for appropriate cellular function [81]. A limited number of genes that corresponded with regulation of these cellular growth pathways were higher in mRNA abundance in the blastocysts resulting from oocytes exposed to greater follicle maturity follicular fluid treatment during IVM (Fig. 5). Alternatively, there were more numerous genes related to positive regulation of cellular growth pathways that were greater expressed in blastocysts originating from oocytes in the lesser follicle maturity treatment (Fig. 5). Due to a variety of growth stimulating pathways and regulatory functions of genes differentially expressed in greater and lesser follicle maturity blastocysts, conclusions related to an effect of follicle maturity treatment during oocyte maturation on resulting blastocyst molecular blueprint for cellular proliferation should be cautiously interpreted. It is interesting to speculate that regulation of cellular division may be more functional in blastocysts of the greater follicle maturity treatment. In support of this hypothesis, *TFPI* was higher in abundance in the greater follicle maturity blastocysts. The protein encoded by this gene may provide a controlling factor on uninhibited cellular growth that is often represented in cancers. Increased expression of *TFPI* has been linked with inhibited ERK/p38 MAPK signaling and results in inhibition of cellular proliferation [82]. This may coincide with an increased abundance of *GSN* in the blastocysts originating from oocytes of the greater follicle maturity treatment. As an important factor for embryo development, the protein GSN exhibits a protective role on cells from excitotoxic death [83]. The higher abundance of both *TFPI* and *GSN* in blastocysts from the greater follicle maturity treatment could indicate increased regulatory mechanisms of simultaneous prevention of excessive cellular growth and disproportionate apoptosis during embryo development. The fact that a far greater number of genes related to cellular proliferation were more abundant in lesser follicle maturity blastocysts suggests that cellular growth and proliferation may be less regulated in such samples. Therefore, we speculate that embryos resulting from maturation with follicular fluid from lesser maturity follicles may be overcompensating for nutrient deficiency during oocyte maturation.

Something noteworthy regarding the cell growth stimulatory genes higher in expression in blastocysts derived from oocytes supported by follicular fluid from preovulatory follicles of lesser maturity is their connections with a multitude of cancers. For instance, studies have demonstrated involvement of *ARRB1*, *MGST1*, and *TTPAL* in colorectal cancer, leading to increased cancer cell proliferation and subsequent poor prognosis [84–86]. Increased abundance of *ARRB1*, as well as *FBXO11*, *LYZ*, *RPS6KB1*, and *SYNGR2* have comparable oncogenic effects in hepatocellular carcinoma [87–91], and *MGST1* and *RPS6KB1* have noted oncogenic roles in various lung cancers [92, 93]. Recent studies have also focused on tumor promotion in gastric cancer and potential targets for treatment, including *MGST1*, *TTPAL*, *FBXO11*, and *IL2RG* [94–98]. Furthermore, additional genes including *LOC100850276*, *LOC101902691*, *LOC101902918*, *LOC104970913 (*also known as *ZNF37A)*, and *LOC517884* have been linked to cellular stress, tumorigenesis, cellular invasion, and inhibition of apoptosis [99–103].

It is essential to establish the similarities between cancer cells and developing embryos. For example, both require a level of increased cellular growth and differentiation. However, an important distinction lies with the amount of control regarding these cellular processes. Put simply, cancer is defined as a group of cells that display uncontrolled cellular growth [89] while successful embryo development relies on controlled and organized mechanisms. Additionally, the present results that suggest higher functionality in greater follicle maturity blastocysts, as well as the need for regulated control of signaling pathways such as Wnt/B-catenin, are essential for embryo development. Alternatively, dysregulation of these pathways has been linked to multiple cancers [104–106]. The differentially expressed genes with higher mRNA abundance in blastocysts from the lesser follicle maturity treatment that demonstrate positive impacts on cellular growth pathways, as well as inefficient utilization of cellular energetics, could be further representation of increased, yet disorganized growth. This said, any conclusions from the current dataset related to dysregulated cell division should be cautiously interpreted due to the varied cell types and functions of blastocysts of the stage utilized in the current study.

Such potential conclusions are exciting, though, as previous studies suggest increased cell division in embryos from oocytes matured in lesser maturity follicular fluid [11–12, 21], and transcriptome profiles reported in the current study create a compelling argument for altered metabolism and cell signaling pathways in these embryos. Aspartate β-hydroxylase is an excellent example of a gene that ties these concepts together. This gene had higher mRNA abundance in blastocysts from the lesser follicle maturity treatment. Although we found its metabolic roles in decreased mitochondrial function, lower ATP, and increased intracellular ROS the most compelling, its protein has been associated with cancer phenotype as a factor promoting cell motility and invasion in pancreatic development [107], and it is upregulated by MAPK and PI3K/AKT, and Wnt/β-catenin signaling to initiate tumor development [108–109].

## Conclusions

The current study was conducted to test the hypothesis that supplementing in vitro maturing oocytes with follicular fluid deriving from preovulatory follicles of greater or lesser physiological maturity would impact the transcriptome of resulting blastocysts. All embryos utilized in this study were of similar morphology, being quality grade 1 and of the expanding or expanded blastocyst stage of development. Nevertheless, RNA-sequencing of these morphologically similar and high-quality grade embryos provides compelling evidence that the intrafollicular milieu of preovulatory follicles of greater versus lesser maturity, when applied to maturing oocytes, leads to differences in the transcriptome of resulting embryos that could impact further embryo development and pregnancy. Differences in follicular fluid support of the maturing oocyte led to molecular signatures of blastocysts that suggest improved metabolism and Wnt/β-catenin signaling in blastocysts originating from oocytes in the greater follicle maturity treatment and dysregulated cell division combined with oxidative stress in the lesser follicle maturity treatment. Future efforts to determine physiological impacts of follicle status on the developing embryo and reveal specific mechanisms that allowed differences in oocyte maturation environment to reflect in the transcriptome of blastocysts are next steps to explore the hypotheses that this study suggests.

## Electronic supplementary material

Below is the link to the electronic supplementary material.

Additional file 1

Additional file 2

## Data Availability

The datasets generated and/or analyzed during the current study are available in the Gene Expression Omnibus database at https://www.ncbi.nlm.nih.gov/geo/, and can be accessed with the accession number GSE279311.

## Abbreviations

COC: cumulus-oocyte complex
CPM: counts per million
DAVID: Database for Annotation, Visualization, and Integrated Discovery
Dvl: disheveled
E2: estradiol
E2: P4:estradiol to progesterone
eFDR: empirical false discovery rate
FDR: false discovery rate
FSH: follicle stimulating hormone
GnRH: gonadotropin releasing hormone
GO: gene ontology
hpi: hours post in vitro fertilization
IETS: International Embryo Technology Society
IVF: in vitro fertilization
IVM: in vitro maturation
JAK/STAT: Janus-activated kinase/signal transducer and activator of transcription
KSOM: potassium supplemented simplex optimized medium
LH: luteinizing hormone
MAPK: mitogen-activated protein kinase
MTOR: mammalian target of rapamycin
OMM: oocyte maturation media
OPLS: orthogonal partial least squares
PI3K/AKT: phosphoinositide 3-kinase/v-AKT murine thymoma viral oncogene homolog 1
PGF: prostaglandin F2 alpha
PZ: putative zygote
ROS: reactive oxygen species
TSG: tumor suppressor genes
