# Supplementary material for: Transcriptome profiles of blastocysts originating from oocytes matured in follicular fluid from preovulatory follicles of greater or lesser maturity
Source: BMC Genomics. 2025 Apr 4;26:339. doi: 10.1186/s12864-025-11521-0 (PMC11969919; doi:10.1186/s12864-025-11521-0)
Supplement: Supplementary file 1 — Additional file 1 [file 12864_2025_11521_MOESM1_ESM.pdf]

Additional file 1: Summary of sequenced reads from blastocyst pools from greater and lesser follicle maturity treatments.

Sample	Follicle Maturity	Raw Sequences	Trimmed Sequences	Mapped Reads	Assigned to Gene	Alignment %	Gene %
**1**	Lesser	27973594	27445045	26997892	22883655	96.5	84.8
**2**	Lesser	31266411	30657925	29804327	24489415	95.3	82.2
**3**	Greater	34388020	33819801	33186191	27743126	96.5	83.6
**4**	Lesser	18639410	18313855	17840072	14761567	95.7	82.7
**5**	Lesser	37285615	36709427	36120347	30110790	96.9	83.4
**6**	Lesser	38180243	37536876	36950294	30372304	96.8	82.2
**7**	Greater	36541576	35938259	35391720	30026795	96.9	84.8
**8**	Greater	25084005	24688105	24383047	20982622	97.2	86.1
**9**	Lesser	26464916	26040359	25603900	21612725	96.7	84.4
**10**	Greater	35087587	34384455	33693510	27914464	96.0	82.8
**11**	Lesser	37740469	37163840	36585900	30649173	96.9	83.8
**12**	Lesser	57471915	56439520	55430000	45280190	96.4	81.7
**13**	Greater	38453153	37855630	37355803	31593872	97.1	84.6
**14**	Lesser	14530538	14265527	14013484	11746287	96.4	83.8
**15**	Greater	32566357	32029735	31611383	26690541	97.1	84.4
**16**	Lesser	13355022	13125550	12852147	10760499	96.2	83.7
**17**	Lesser	40748361	39564996	38880490	32336626	95.4	83.2
**18**	Greater	19123042	18810491	18527112	15693492	96.9	84.7
**19**	Greater	21402942	20925405	20477684	16777664	95.7	81.9
**20**	Lesser	20728882	20286513	19878204	16282603	95.9	81.9
**21**	Lesser	19443231	19078845	18806993	15884487	96.7	84.5
**22**	Greater	18235467	17935331	17574415	14252223	96.4	81.1
**23**	Lesser	26961100	26352609	25772365	21165414	95.6	82.1
**24**	Greater	55530730	54288179	53479288	44923647	96.3	84.0
**25**	Lesser	28469467	27932660	27506800	22821489	96.6	83.0
**26**	Greater	17524554	17206547	16924114	14191919	96.6	83.9
**27**	Greater	23624195	23222179	22870384	19154343	96.8	83.8
	**Mean**	**29511882**	**28963617**	**28463625**	**23744516**	**96.4**	**83.4**
