# Supplementary material for: Transcriptome profiles of blastocysts originating from oocytes matured in follicular fluid from preovulatory follicles of greater or lesser maturity
Source: BMC Genomics. 2025 Apr 4;26:339. doi: 10.1186/s12864-025-11521-0 (PMC11969919; doi:10.1186/s12864-025-11521-0)
Supplement: Supplementary file 2 — Additional file 2 [file 12864_2025_11521_MOESM2_ESM.pdf]

Additional file 2 Differentially expressed genes between blastocyst pools from greater and lesser follicle maturity treatments.

Official Gene Symbol	Gene Name or Description	eFDR (edgeR)	eFDR (DESeq2)	Mean Abundance (Greater)	Mean Abundance (Lesser)
Upregulated in blastocysts produced from oocytes matured in the presence of follicular fluid from preovulatory follicles of greater maturity					
ADAT3	adenosine deaminase tRNA specific 3	0.004	0.005	8.399	7.025
ALDH7A1	aldehyde dehydrogenase 7 family member A1	0.010	0.009	208.315	162.773
ARF5	ADP ribosylation factor 5	0.007	0.010	78.644	65.373
AXIN2	axin 2	0.005	0.005	11.812	7.956
C14H8orf89	chromosome 14 C8orf89 homolog	0.011	0.007	32.762	26.077
CPNE9	copine family member 9	0.009	0.010	1.421	0.745
DBT	dihydrolipoamide branched chain transacylase E2	0.007	0.007	19.399	13.942
DYNC2I2	dynein 2 intermediate chain 2	0.004	0.004	31.359	25.536
ENTPD6	ectonucleoside triphosphate diphosphohydrolase 6	0.013	0.012	15.674	11.738
FAM117A	family with sequence similarity 117 member A	0.011	0.005	48.250	42.915
FZD6	frizzled class receptor 6	0.012	0.012	16.934	13.197
GSN	gelsolin	0.002	0.003	233.212	152.016
HNRNPLL	heterogeneous nuclear ribonucleoprotein L like	0.005	0.005	75.341	62.075
IDH3A	isocitrate dehydrogenase (NAD(+)) 3 catalytic subunit alpha	0.002	0.004	565.167	474.123

IP6K1	inositol hexakisphosphate kinase 1	0.011	0.009	11.456	8.254
KIF3B	kinesin family member 3B	0.007	0.009	2.735	1.487
LOC112442296	uncharacterized LOC112442296	0.010	0.011	2.244	1.789
LOC516378	intestinal alkaline phosphatase VII	0.004	0.005	337.700	264.927
LOC526041	ankyrin repeat domain protein	0.009	0.009	5.612	3.727
LOC528262	intestinal-type alkaline phosphatase	0.002	0.004	336.733	271.627
LYRM4	LYR motif containing 4	0.005	0.007	82.997	66.905
NIPSNAP2	nipsnap homolog 2	0.013	0.006	160.309	146.742
NOTCH4	notch receptor 4	0.003	0.006	1.451	0.949
PEX10	peroxisomal biogenesis factor 10	0.009	0.007	9.752	7.347
SLC15A1	solute carrier family 15 member 1	0.002	0.003	24.706	16.070
SLC25A15	solute carrier family 25 member 15	0.011	0.011	32.765	24.805
SNTB1	syntrophin beta 1	0.013	0.014	23.502	17.857
STARD9	StAR related lipid transfer domain containing 9	0.002	0.003	5.133	3.655
TCEANC2	transcription elongation factor A N-terminal and central domain containing 2	0.011	0.007	10.143	8.657
TFPI	tissue factor pathway inhibitor	0.013	0.012	24.439	19.345
TMEFF1	transmembrane protein with EGF like and two follistatin like domains 1	0.004	0.004	2.733	1.746

TMEM198B	transmembrane protein 198B	0.013	0.013	1.669	0.961
TMEM39A	transmembrane protein 39A	0.001	0.002	30.293	23.780
TNFRSF8	TNF receptor superfamily member 8	0.001	0.003	9.334	5.926
TPRN	taperin	0.013	0.008	14.175	10.774
UEVLD	UEV and lactate/malate dehyrogenase domains	0.003	0.004	22.589	16.539
ZNF398	zinc finger protein 398	0.013	0.014	1.913	1.368
Upregulated in blastocysts produced from oocytes matured in the presence of follicular fluid from preovulatory follicles of lesser maturity					
ACAD10	acyl-CoA dehydrogenase family member 10	0.009	0.009	1.871	2.912
AICDA	activation induced cytidine deaminase	0.004	0.007	2.545	5.605
ANK3	ankyrin 3	0.002	0.005	2.819	4.864
ANKRD31	ankyrin repeat domain 31	0.004	0.004	5.241	7.918
ARRB1	arrestin beta 1	0.011	0.011	77.877	102.782
ASPH	aspartate beta-hydroxylase	0.003	0.005	20.188	27.124
BLA-DQB	MHC class II antigen	0.008	0.011	2.777	4.983
CASP8AP2	caspase 8 associated protein 2	0.008	0.008	34.815	43.445
CD52	CD52 molecule	0.002	0.004	11.976	19.425
CSNK1B	casein kinase I beta	0.007	0.008	12.240	19.627

DHRS9	dehydrogenase/reductase 9	0.009	0.007	9.658	19.374
DMRT2	doublesex and mab-3 related transcription factor 2	0.003	0.007	0.749	1.529
DNAJC22	DnaJ heat shock protein family (Hsp40) member C22	0.003	0.005	3.207	7.644
DOCK11	dedicator of cytokinesis 11	0.003	0.004	13.567	23.346
DUXA	double homeobox A	0.005	0.008	7.187	17.270
EIF2AK2	eukaryotic translation initiation factor 2 alpha kinase 2	0.007	0.008	3.537	7.153
FBXO11	F-box protein 11	0.014	0.012	4.711	6.095
GPATCH2L	G-patch domain containing 2 like	0.006	0.004	15.054	19.237
H2BC18	H2B clustered histone 18	0.002	0.004	1.312	3.164
HHAT	hedgehog acyltransferase	0.012	0.008	1.946	2.775
HHEX	hematopoietically expressed homeobox	0.006	0.009	3.758	5.762
IL2RG	interleukin 2 receptor subunit gamma	0.009	0.010	2.419	4.759
JCHAIN	joining chain of multimeric IgA and IgM	0.009	0.010	3.904	5.632
KIAA1671	KIAA1671	0.006	0.005	8.372	11.600
KIF27	kinesin family member 27	0.001	0.003	7.504	11.446
KLF3	KLF transcription factor 3	0.012	0.007	19.242	23.876
LOC100140788	zinc finger protein 596-like	0.002	0.003	1.438	5.181
LOC100847802	uncharacterized LOC100847802	0.008	0.013	3.703	7.033

LOC100850276	UL16-binding protein 3	0.010	0.011	2.981	5.653
LOC101902691	uncharacterized LOC101902691	0.013	0.010	3.181	7.559
LOC101902754	cytochrome b-c1 complex subunit Rieske, mitochondrial-like	0.001	0.003	14.646	26.591
LOC101902918	zinc finger CCCH domain-containing protein 15-like	0.008	0.011	1.054	1.557
LOC101903765	UL16-binding protein 3-like	0.001	0.002	20.214	31.251
LOC101903925	uncharacterized LOC101903925	0.002	0.003	2.696	6.014
LOC101905019	uncharacterized LOC101905019	0.010	0.011	1.552	2.671
LOC101908683	uncharacterized LOC101908683	0.008	0.006	1.496	2.881
LOC104970913	zinc finger protein 37A	0.005	0.009	8.110	11.003
LOC107132012	EKC/KEOPS complex subunit LAGE3-like	0.010	0.006	0.865	4.009
LOC112441492	zinc finger and SCAN domain containing 4	0.004	0.006	6.052	10.909
LOC112442848	U2 spliceosomal RNA	0.003	0.004	1.141	2.449
LOC112444653	5.8S ribosomal RNA	0.002	0.004	81.143	113.303
LOC112444864	uncharacterized LOC112444864	0.002	0.004	1.296	1.953
LOC112448649	U6 spliceosomal RNA	0.003	0.008	0.742	1.381
LOC132342561	uncharacterized LOC132342561	0.011	0.013	1.137	2.214
LOC132344302	uncharacterized LOC132344302	0.003	0.006	1.627	4.073

LOC508666	C-C motif chemokine 23	0.011	0.012	27.985	40.721
LOC517884	PRAME family member 12	0.001	0.002	14.016	22.405
LOC618696	trophoblast Kunitz domain protein 1	0.005	0.008	26.124	58.319
LYZ	lysozyme (renal amyloidosis)	0.012	0.014	2.541	4.071
MARF1	meiosis regulator and mRNA stability factor 1	0.008	0.013	5.857	8.899
MGC137030	uncharacterized protein MGC137030	0.007	0.011	8.091	18.409
MGST1	microsomal glutathione S-transferase 1	0.007	0.009	37.612	74.188
MYL4	myosin light chain 4	0.011	0.008	4.198	8.876
NR3C2	nuclear receptor subfamily 3 group C member 2	0.009	0.013	1.157	2.814
PCDH17	protocadherin 17	0.008	0.011	1.151	2.543
PHKA1	phosphorylase kinase regulatory subunit alpha 1	0.013	0.012	9.454	13.530
POMC	proopiomelanocortin	0.010	0.012	28.023	36.134
PRELID3A	PRELI domain containing 3A	0.013	0.012	1.538	2.522
PRICKLE1	prickle planar cell polarity protein 1	0.010	0.012	1.276	2.469
PRSS2	serine protease 2	0.001	0.003	0.700	1.919
RBM15	RNA binding motif protein 15	0.008	0.006	11.693	15.312
RBM18	RNA binding motif protein 18	0.005	0.007	26.114	34.141
RPS6KB1	ribosomal protein S6 kinase B1	0.011	0.013	26.022	30.784

S100A13	S100 calcium binding protein A13	0.001	0.002	2.760	4.929
SEC16B	SEC16 homolog B, endoplasmic reticulum export factor	0.005	0.012	1.026	1.876
SFXN5	sideroflexin 5	0.011	0.009	4.258	5.886
SIGLECL1	SIGLEC family like 1	0.013	0.009	1.985	3.552
SYNGR2	synaptogyrin 2	0.013	0.013	18.419	23.834
THAP5	THAP domain containing 5	0.012	0.013	31.743	40.334
TKDP1	trophoblast Kunitz domain protein 1	0.008	0.006	20.184	72.089
TTLL5	tubulin tyrosine ligase like 5	0.006	0.006	5.602	8.517
TTPAL	alpha tocopherol transfer protein like	0.011	0.014	3.758	6.721
TUBA4A	tubulin alpha 4a	0.008	0.010	6.186	10.631
VAMP5	vesicle associated membrane protein 5	0.005	0.007	1.125	2.806
VAMP9	vesicle associated membrane protein 9	0.010	0.008	0.822	2.287
WDR7	WD repeat domain 7	0.013	0.008	4.244	5.766
